# Stereotactic irradiation of non-small cell lung cancer brain metastases: evaluation of local and cerebral control in a large series

**DOI:** 10.1038/s41598-020-68209-6

**Published:** 2020-07-08

**Authors:** Etienne Fessart, Raphaëlle Mouttet Audouard, Florence Le Tinier, Bernard Coche-Dequeant, Thomas Lacornerie, Emmanuelle Tresch, Arnaud Scherpereel, Eric Lartigau, Xavier Mirabel, David Pasquier

**Affiliations:** 1 Academic Department of Radiation Oncology, Centre Oscar Lambret, 59020 Lille Cedex, France; 2 Department of Biostatistics, Centre Oscar Lambret, Lille, France; 3 Department of Pulmonary and Thoracic Oncology, University of Lille, University Hospital (CHU), Lille, France; 4 CRISTAL UMR CNRS 9189, Lille University, Lille, France

**Keywords:** Cancer, Oncology, Metastasis

## Abstract

Stereotactic radiotherapy (SRT) of brain metastases (BM) results are often reported in the heterogeneous primitive population. Here, we report our experience in consecutively treated patients who underwent SRT alone for BM from non-small cell lung cancer (NSCLC). This retrospective analysis included consecutive patients with no history of cerebral treatment who underwent Cyberknife™ SRT for BM from NSCLC in our institution from 2007 to 2016. One hundred patients were included in the study, with a median follow-up of 33 months (20–64). Mean age was 63 years (SD ± 10); 88% had Karnofsky Performance Status (KPS) > 70; 67% had unique BM; 18 patients received single-fraction SRT (20–25 Gy), and 82 received hypo-fractionated SRT (HSRT) (24–36 Gy in 3–5 fractions). We reported a complication rate of 17% (2% of G3-4). Median survival was 10.1 months [95% confidence interval (CI) 7.8–13.9]. At 1 year, local and cerebral control rates were respectively 78.7% (95% CI 70–86.5%) and 43% (95% CI 33.5–53%). Thirty patients underwent salvage treatment (whole brain radiation therapy, n = 13; SRT, n = 14; surgery, n = 3). Cyberknife™-based SRT is an effective treatment associated with high local control rate with low morbidity for patients with NSCLC’s BM. Close follow-up is necessary to perform salvage treatment.

## Introduction

Lung cancer brain metastases (BM) are a common diagnosis and source of morbidity. Of the 1.82 million new cases of lung cancer diagnosed annually in the world, more than 30% present BM (including 20% initially and up to 80% throughout their disease course)^1, 2^. Non-small cell lung cancer (NSCLC) is the most common histology (85% of cases), with adenocarcinoma predominating. Patients with BM have a poor prognosis, with a median survival of 3–15 months, according to the diagnosis-specific graded prognostic assessment (DS-GPA), including Karnofsky Performance Status (KPS), age, number of metastases, and presence of extracranial metastasis^3^. More recently, adenocarcinoma patient groups with epidermal growth factor receptor (EGFR) or anaplastic lymphoma kinase (ALK) alteration have been shown to have a median survival of up to 47 months^4^, showing the efficacy of new systemic targeted treatments.

The management of patients with BM has evolved from whole-brain radiotherapy (WBRT) alone to combinations of locally directed therapies, including surgical resection and/or stereotactic radiotherapy (SRT) with or without WBRT. Randomized trials including patients with limited numbers of BM (≤ 5) from various primitive cancers have shown a better local control for SRT but no differences in overall survival between these different modalities for the whole population^5^. Sub-group analysis or meta-analysis has found that SRT may improve survival for younger patients (< 50 years old), solitary BM, and GPA > 2^6–8^. Adding WBRT to SRT reduces the development of distant BM, but it comes at the cost of cognitive toxicity^9, 10^.

There are only retrospective data evaluating the results of SRT in a homogenous population of BM from NSCLC^11, 12^, showing a good local control at 1 year (80–90%) and distant brain control (60–80% ) rates after Gammaknife™ (GK) procedure. The goal of our study was to evaluate our practice for SRT with Cyberknife™ (CK) in this population.

## Methods and materials

### Data acquisition

Patients’ agreement before using their data was required. Informed consent was obtained for all patients. The Institutional Committee on Human Research of Centre Oscar Lambret (Lille) has approved this retrospective study. All research was performed in accordance with relevant regulations.

Using the local database, we retrospectively studied the medical records of all patients aged 18 or over who underwent SRT by CK for the treatment of all of their NSCLC BM in our institution between 2007 and October 2016. All patients with previous history of cerebral treatment (surgery or radiotherapy) were excluded.

All lesions detected by magnetic resonance imaging (MRI) were irradiated by Cyberknife™. Baseline characteristics, treatment modalities, acute toxicities (CTCAE v4), clinical and radiological outcome every 3 months, and salvage therapy data were recorded for each patient. BM were considered metachronous if time from primary diagnosis was > 6 months.

### Statistical analysis

Clinical and treatment characteristics were described by median, range, mean, and standard error for continuous variables and by frequency and percentages for categorical variables. Treatment response was first estimated using the response evaluation criteria in solid tumors (RECIST) criteria, and then using the response assessment criteria for brain metastases (RANO-BM) criteria when clinical status was available. RANO-BM has been developed to avoid equating treatment effect with tumor progression. Indeed, it takes into account clinical assessment (neurological examination), corticosteroid dosing, and needs a 6–12 weeks imaging confirmation to assess progression.

Survival endpoints were computed from the date of the end of radiotherapy. Overall survival was estimated as time interval to death using Kaplan–Meier method, and patients alive were censored at the date of last news. Time to local progression (local control), time to cerebral progression (cerebral progression away from the treated site), time to neurological deterioration, and time to salvage treatment were estimated as time interval using the competing risk method (Kalbleisch and Prentice).

Associations between clinical or treatment factors and survival endpoints were analyzed using Cox proportional hazard regression models for overall survival, and using Fine and Gray models for time to cerebral progression, time to local progression, and time to neurological deterioration. Variables associated with survival with a significance level p < 0.10 in univariate analysis were included in multivariate regression models. A backward stepwise selection of variables was performed if the number of variables to include in the model was too large compared to the number of observed events (at least 10 events should be observed by variable). The significance level was set to p < 0.05. The statistical software used was Stata v13.1 (StataCorp, 2013. Stata Statistical Software: Release 13. College Station, TX: StataCorp, LP).

## Results

### Patient and treatment characteristics

One hundred patients were included between 2007 and 2016. The mean follow-up period was 33 months (range 20–64 months). Patients’ demographics and clinical data are summarized in Table 1. Mean age was 63 years (SD ± 10 years); sex ratio was 3.3 men for 1 woman. KPS at baseline was 90–100, 70–80, and < 70 for 30%, 58%, and 12%, respectively. Forty-two percent of patients presented with extra-cranial metastasis. Forty-four percent were symptomatic for their BM. Sixty-seven percent had a solitary BM, 23% had 2 BM, and 10% had 3 or more BM. Mean long axis of BM was 16.6 mm (± 10.4 mm). Mean time from simulation and diagnosis MRI to treatment was, respectively, 18.1 ± 12.1 days and 27.7 ± 22.5 days. SRT was conducted with Cyberknife™. The gross tumor volume (GTV) was outlined on both enhanced contrast CT images and CT-MRI fused images. Planning target volumes (PTVs) were determined by expansion of the GTV with a 1 mm margin. The most frequent schedules were 27 Gy in 3 fractions (69%), 18–25 Gy in 1 fraction (18%), and 30 Gy in 5 fractions (9%), prescribed on isodose 80%.

**Table 1:** Patients and treatment characteristics.

Characteristics (N = 100)	No. patients	%
**Age**		
< 60 years	38	38.0%
60–69 years	34	34.0%
≥ 70 years	28	28.0%
**Sex**		
Men	77	77.0%
Women	23	23.0%
**KPS**		
90–100	30	30.0%
70–80	58	58.0%
< 70	12	12.0%
**Extra cranial metastasis**	42	42%
**DS-GPA**		
0–1	9	9.0%
1.5–2	42	42.0%
2.5–3	41	41.0%
3.5–4	7	7%
**RPA**		
1	28	28.0%
2	59	59.0%
3	12	12.0%
**Histology of primitive tumor**		
Adenocarcinoma	69	69.0%
SCC	23	23.0%
Other	8	8.0%
**Number of previous chemotherapy lines (N = 98)**		
0	47	48.0%
1	32	32.7%
≥ 2	19	19.4%
**Primary tumor control before SRT (N = 99)**		
No	22	24.2%
Yes	45	45.5%
Synchronous diagnosis	30	0.3%
**Clinical signs**		
Non symptomatic	54	54%
Symptomatic	44	44.0%
Deficit	26	26.0%
Seizure	13	13.0%
**Corticotherapy**	41	45.6%
**Synchronous or metachronous BM**		
Synchronous	32	32%
Metachronous	68	68%
**No. lesions**		
1	67	67.0%
2	23	23.0%
≥ 3	10	10.0%
**Longest diameter (mm) by tumor**		
Median (range)	14.8	(2–46)
Mean (standard deviation)	16.6	10.4
**Tumor localization**		
Posterior fossa (+ /- other)	23	23%
No posterior fossa	77	77%
**Time MRI-treatment (days)**		
Mean (standard deviation)	27.7	22.5
**Time simulation-treatment (days)**		
Mean (standard deviation)	18.1	12.1
**Treatment schedule**		
27 (Gy)/3 fractions	69	69.0%
18–25 (Gy)/1 fraction	18	18.0%
30 (Gy)/5 fractions	9	9.0%
Other	4	4.0%

*KPS* Karnofsky performance status, *DS-GPA* diagnosis-specific graded prognostic assessment, *RPA* recursive partitioning analysis, *SCC* squamous cell carcinoma, *BM* brain metastases, *MRI* magnetic resonance imaging.

### Survival

**Figure 1:**
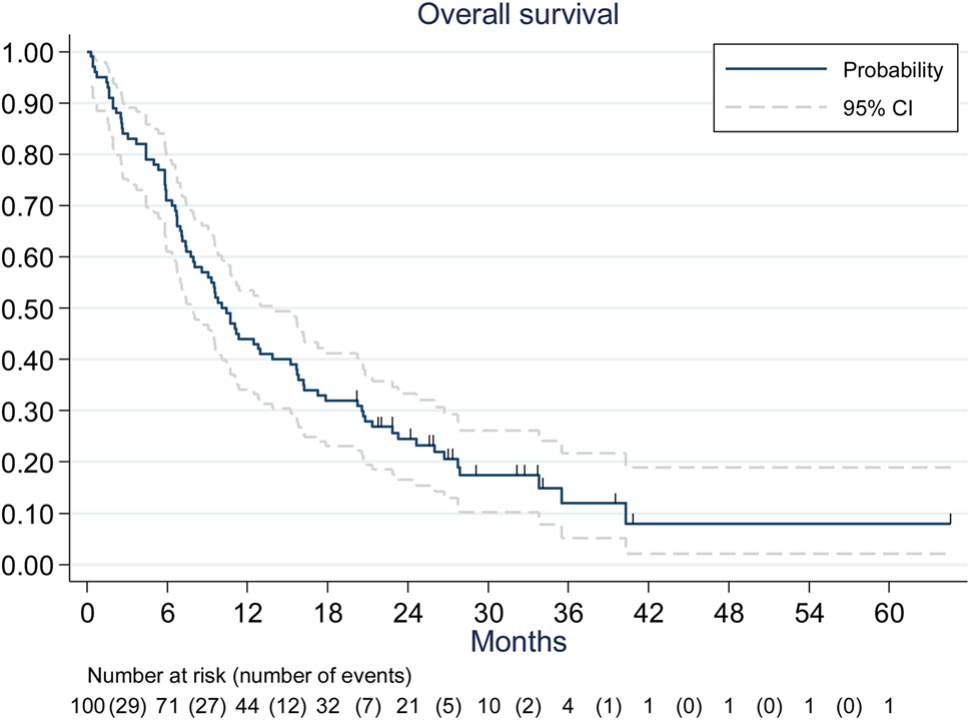
Overall survival.

Median overall survival was 10.1 months [95% confidence interval (CI) 7.8–13.9 months]. One-year overall survival rate was 44% (95% CI 34.1–53.4%) (Fig. 1). Using univariate analysis, the major prognostic factors were the KPS (p = 0.031), the recursive partitioning analysis (RPA) class (p = 0.001), and the GPA score (p = 0.0001). The multivariate analysis found that patients with a high GPA score had statistically better survival (p = 0.01), with a HR = 0.22 (95% CI 0.07–0.74) for GPA = 3.5–4.

### Local and cerebral progression

**Figure 2:**
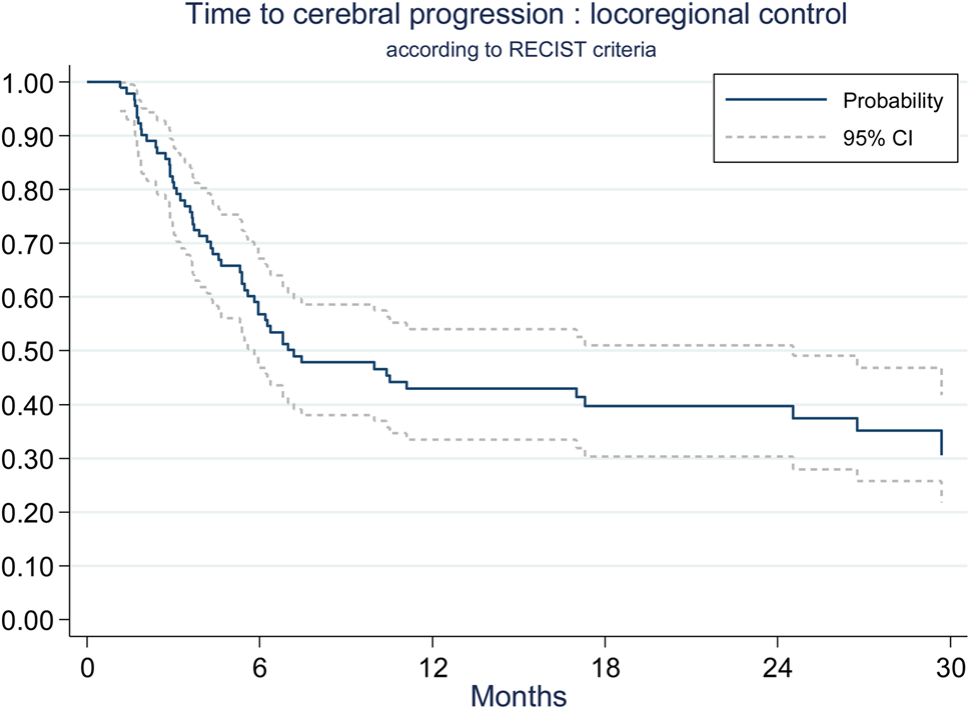
Loco-regional control.

**Figure 3:**
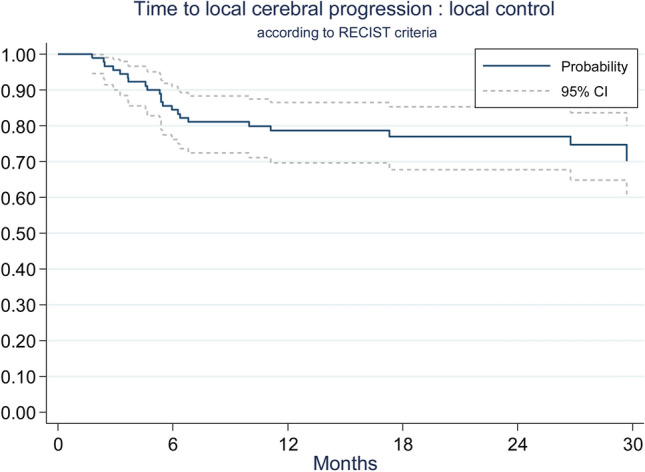
Local control.

Time to cerebral and local progression were similar according to RECIST 1.1 (n = 91) and to RANO-BM (n = 89) evaluations (Table 2). We chose to report RECIST 1.1 results because more data were available. At 1 year, using the competing risk method, cerebral control was 43% (95% CI 33.5–53%) (Fig. 2), and local control was 78.7% (95% CI 70–86.5%) (Fig. 3).

**Table 2:** Time to cerebral and to local progression.

Characteristics	RECIST 1.1 (N = 91)		RANO-BM (N = 89)	
**Time to cerebral progression**^a^				
Cumulative incidence of cerebral progression at 6 months (%) (95% CI)	43.2%	(32.9–53.1)	46.5%	(35.8–56.5)
Cumulative incidence of cerebral progression at 1 year (%) (95% CI)	57.0%	(46.1–66.5)	60.6%	(49.5–70.0)
**Time to local progression**^b^				
Cumulative incidence of local progression at 6 months (%) (95% CI)	15.6%	(9.0–23.8)	15.9%	(9.2–24.3)
Cumulative incidence of local progression at 1 year (%) (95% CI)	21.3%	(39.1–59.5)	21.9%	(13.9–31.0)

^a^Time to cerebral progression: death before failure is a competing risk event.

^b^Time to local progression: death or cerebral progression before local failure is a competing risk event.

There were no factors associated with cerebral control at 1 year using univariate and multivariate analysis. We observed a trend towards a worse cerebral control in tumors located in the posterior fossa (p = 0.06 in univariate and multivariate analysis).

Using univariate analysis, increasing number of BM (p = 0.004), metachronous BM (p = 0.041), and increasing time between simulation and treatment (p = 0.04) were associated with a lower local control. Using multivariate analysis, the increasing number of brain metastasis [subhazard ratio (SHR) = 2 (95% CI 1.4–2.87)] and metachronous BM [SHR = 7.46 (95% CI 1.58–1.01)] remained associated with a lower local control at 1 year. The acute toxicity rate was 17%, including 2% of G3–4 (fatigue and cerebral edema). Late toxicity was not reported due to missing data.

### Time to neurological deterioration

For the 96 patients with neurologic assessment data available in follow-up, cumulative incidence of neurological deterioration at 1 year was 18.1% (95% CI 11.1–36.5%). Using univariate and multivariate analysis, factors associated with a lower neurological deterioration were adenocarcinoma histology (p = 0.001 in multivariate) and solitary BM (p < 0.001 in multivariate).

### Salvage therapy

**Figure 4:**
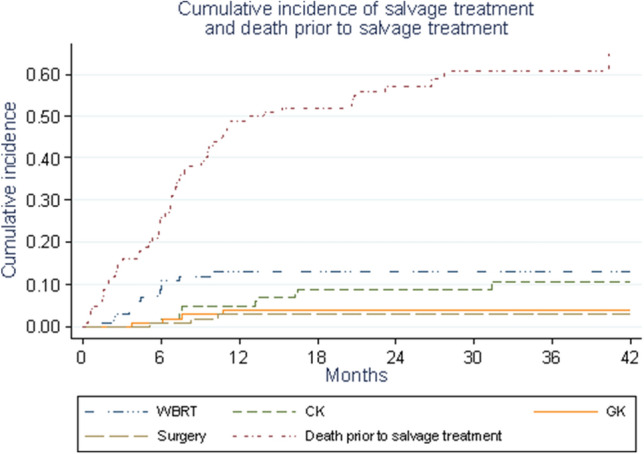
Salvage therapy.

At 1 year, 22 and 56 local and cerebral progressions have been reported according to RECIST respectively. After initial SRT, a total of 30 patients received salvage therapy: 14 received salvage SRT, 13 received salvage WBRT, and 3 underwent salvage surgery as their first salvage treatment (Fig. 4). Sixty patients died without requiring a second brain treatment.

## Discussion

SRT has gained increasing relevance within the therapeutic armamentarium of BM. Our retrospective study is one of the largest series reporting SRT results for BM of NSCLC without previous history of cerebral treatment. Well-known prognosis scores, such as RPA^12^ and DS-GPA^3^ were confirmed. Nevertheless, our median survival was higher than those predicted by these prognostic scores: for example, RPA 1 class median survival was 20.7 months (95% CI 15.2–33.8 months) in our study, while 7.1 months were reported in Gaspar’s study for patients treated in the 1980s^12^. This illustrates the global care improvement for BM of NSCLC. The more recent Lung-molGPA score, described by Sperduto et al., takes into account histology and EGFR/ALK alterations and shows survival of up to 47 months for adenocarcinomas with a high score^4^. For example in Fan et al. patients with EGFR-mutant adenocarcinoma and BM treated with icotinib exhibited prolonged survival, and a longer duration of cerebral control was observed with brain radiotherapy^13^. In a multi-institutional analysis Magnuson et al. demonstrated that the use of upfront EGFR-tirosyne kinase inhibitor (TKI), and deferral of radiotherapy, is associated with inferior overall survival in patients with EGFR-mutant NSCLC who develop brain metastases. Radiosurgery followed by EGFR-TKI resulted in the longest overall survival and allowed patients to avoid the potential neurocognitive sequelae of WBRT^14^. The optimal combination of SRT and the several TKI has not yet been determined. It would have been interesting for our population treated between 2007 and 2016 because some of them may have been treated with anti-EGFR or immunotherapy. However, genetic alterations were not determined in most of our patients due to the recruitment time.

Our overall survival, local and cerebral control rates, and acute toxicities are in agreement with the literature (Table 3). In the published studies, mainly retrospectives, median survival is approximately 10 months, 1-year local and cerebral control rates are, respectively, 70–90% and 40–60%^7, 11–16^. Only a few retrospective studies have evaluated SRT for homogenous NSCLC’s BM populations. Zairi et al. reported better outcomes, but the population was strictly selected (82% of solitary BM, with limited size), as suggested by the one-year cerebral control rate of 79% versus 43% in our series^11^.

**Table 3:** Comparison of our study with literature.

	Authors	Population	SRT schedule	Median follow-up	Med survival	Local control	Cerebral control	Toxicities	Salvage therapy
100% NSCLC	Bowden 2015^12^ Retrospective	N = 720 nBM = 1 (38%)-19 RPA ≤ 2: 98% Previous WBRT 52%	18-20 Gy/1f ID 50–80% GK	8 months (range 1–124)	8.5 months (95% CI 0.5–158)	80%	58%	NR	24%
	Zairi 2014^11^ Retrospective	N = 89 nBM = 1 (82%)-6 RPA ≤ 2: 78%	18–26 Gy/1f ID 50% GK	28 months (range 1–99)	24 months (95% CI NR)	At 1 year: 91.5%	At 1 year: 79%	Acute: 10% G1-2 0% G3-4	22%
	Our study Retrospective	N = 100 nBM = 1 (67%)-6 DS-GPA ≥ 2.5: 48.5% RPA ≤ 2: 88%	18-36 Gy/1-5f ID 80% CK	33 months (range 20–64)	10.1 months (95% CI 7.8–13.9)	At 1 year: 78.7%	At 1 year: 43%	Acute : 15% G1-2 2% G3	30%

*LC* lung cancer, *UK* unknown, *nBM* number of BM treated by patient, *ID* Isodose, *GK* Gammaknife™, *CK* Cyberknife™, *LINAC* linear accelerator.

Several factors have been demonstrated to influence local tumor control achieved with SRT. These include prescribed radiation dose, lesion size, histology, and lesion morphology (tumor necrosis is associated with a poor response)^17^. A review of retrospective studies showed a local control rate > 70% for a BED_12_ > 40 Gy (for α/β = 12 Gy)^18^. Rodrigues et al. published a predictive score for BM control after SRT, including dose and morphology, based on retrospective data^19^. This score should be validated prospectively. In our study, we calculated BED_12_ of the SRT schedules: only 1 received less than 40 Gy (treated with 24 Gy in 4 fractions). We did not report BM morphology, but we found that adenocarcinoma histology was associated with lower neurological deterioration (p = 0.001), as well as solitary BM (p < 0.001), suggesting that these patients have the best clinical benefit from SRT procedures.

SRT, compared with WBRT, offers the possibility of salvage therapy^15^. In our study, more than half of patients who had a cerebral progression at 1 year (43%) underwent salvage therapy (25% (95% CI 17–33.8%) at 1 year), mainly by WBRT (13% (95% CI 7.3–20.4%) at 1 year) or SRT. However, most patients (n = 60) died without salvage therapy at the time of analysis. This is in agreement with literature. McTyre et al., in a retrospective analysis of 2,657 patients who underwent BM SRT, found a high rate of death without cerebral relapse^16^. Thus, some asymptomatic patients may not benefit from SRT procedures as they die rapidly from extra-cerebral disease. A careful selection of patients should be done before undergoing SRT.

Our series shows good local control after SRT with low acute toxicity; these patients require regular follow-up because of the risk of brain progression. Nevertheless, more than half of the patients did not require a second brain treatment. We have confirmed the prognostic role of the GPA score in multivariate analysis and patients with preserved health status and a limited number of metastases are the best candidates for SRT as recommended in the guidelines^20, 21^.

## Conclusion

While individualized oncologic treatments are becoming a common practice, it is necessary to evaluate BM management in a homogeneous primitive population. In patients with a limited number of BM from NSCLC, SRT is an effective treatment associated with high local control rate, low neurological deterioration, and with low morbidity. Close follow-up with MRI is mandatory to perform salvage treatment. Patients with BM from NSCLC should be included in randomized trials evaluating new systemic therapies, to monitor synergic effects (efficacy or toxicity), and to clarify the role of SRT in the global care of their BM.

## Data Availability

The datasets generated during and/or analysed during the current study are available from the corresponding author on reasonable request.

## References

[1] Ferlay J (2015). Cancer incidence and mortality worldwide: sources, methods and major patterns in GLOBOCAN 2012. Int. J. Cancer..

[2] Ulahannan D, Khalifa J, Faivre-Finn C, Lee SM (2017). Emerging treatment paradigms for brain metastasis in non-small-cell lung cancer: an overview of the current landscape and challenges ahead. Ann. Oncol..

[3] Sperduto PW (2012). Summary report on the graded prognostic assessment: an accurate and facile diagnosis-specific tool to estimate survival for patients with brain metastases. J. Clin. Oncol..

[4] Sperduto PW (2017). Estimating survival in patients with lung cancer and brain metastases: an update of the graded prognostic assessment for lung cancer using molecular markers (Lung-molGPA). JAMA. Oncol..

[5] Soon YY, Tham IWK, Lim KH, Koh WY, Lu JJ (2014). Surgery or radiosurgery plus whole brain radiotherapy versus surgery or radiosurgery alone for brain metastases. Cochrane Database Syst. Rev..

[6] Andrews DW (2004). Whole brain radiation therapy with or without stereotactic radiosurgery boost for patients with one to three brain metastases: phase III results of the RTOG 9508 randomised trial. Lancet.

[7] Sahgal A (2015). Phase 3 trials of stereotactic radiosurgery with or without whole-brain radiation therapy for 1 to 4 brain metastases: individual patient data meta-analysis. Int. J. Radiat. Oncol..

[8] Aoyama H, Tago M, Shirato H (2015). Stereotactic radiosurgery with or without whole-brain radiotherapy for brain metastases: secondary analysis of the JROSG 99-1 randomized clinical trial. JAMA Oncol..

[9] Brown PD (2016). Effect of radiosurgery alone vs radiosurgery with whole brain radiation therapy on cognitive function in patients with 1 to 3 brain metastases: a randomized clinical trial. JAMA.

[10] Chang EL (2009). Neurocognition in patients with brain metastases treated with radiosurgery or radiosurgery plus whole-brain irradiation: a randomised controlled trial. Lancet. Oncol..

[11] Zairi F (2014). Relevance of gamma knife radiosurgery alone for the treatment of non-small cell lung cancer brain metastases. Clin. Neurol. Neurosurg..

[12] Bowden G (2015). Gamma Knife radiosurgery for the management of cerebral metastases from non-small cell lung cancer. J. Neurosurg..

[13] Fan Y, Xu Y, Gong L (2017). Effects of icotinib with and without radiation therapy on patients with EGFR mutant non-small cell lung cancer and brain metastases. Sci Rep..

[14] Magnuson WJ, Lester-Coll NH, Wu AJ (2017). Management of brain metastases in tyrosine kinase inhibitor-naïve epidermal growth factor receptor-mutant non-small-cell lung cancer: a retrospective multi-institutional analysis. J Clin Oncol..

[15] Yamamoto M (2014). Stereotactic radiosurgery for patients with multiple brain metastases (JLGK0901): a multi-institutional prospective observational study. Lancet. Oncol..

[16] McTyre E (2018). Multi-institutional competing risks analysis of distant brain failure and salvage patterns after upfront radiosurgery without whole brain radiotherapy for brain metastasis. Ann. Oncol..

[17] Thiagarajan A, Yamada Y (2017). Radiobiology and radiotherapy of brain metastases. Clin. Exp. Metastasis..

[18] Wiggenraad R, Verbeek-de Kanter A, Kal HB, Taphoorn M, Vissers T, Struikmans H (2011). Dose-effect relation in stereotactic radiotherapy for brain metastases: a systematic review. Radiother. Oncol..

[19] Rodrigues G, Zindler J, Warner A, Lagerwaard F (2013). Recursive partitioning analysis for the prediction of stereotactic radiosurgery brain metastases lesion control. Oncologist..

[20] Planchard D, Popat S, Kerr K (2018). Metastatic non-small cell lung cancer: ESMO Clinical Practice Guidelines for diagnosis, treatment and follow-up [published correction appears in Ann Oncol. 2019 May;30(5):863-870]. Ann Oncol..

[21] National Comprehensive Cancer Network. Central Nervous System Cancers. Version 2.2020. April 30, 2020 https://www.nccn.org/professionals/physician_gls/pdf/cns.pdf. Acceded May 24th 2020

